# Structural basis for selective inhibition of immunoglobulin E-receptor interactions by an anti-IgE antibody

**DOI:** 10.1038/s41598-018-29664-4

**Published:** 2018-08-01

**Authors:** Jiun-Bo Chen, Faruk Ramadani, Marie O. Y. Pang, Rebecca L. Beavil, Mary D. Holdom, Alkistis N. Mitropoulou, Andrew J. Beavil, Hannah J. Gould, Tse Wen Chang, Brian J. Sutton, James M. McDonnell, Anna M. Davies

**Affiliations:** 1 Genomics Research Center, Academia Sinica, Taipei, 115 Taiwan; 2 King’s College London, Randall Centre for Cell and Molecular Biophysics, London, SE1 1UL United Kingdom; 3 Medical Research Council & Asthma UK Centre in Allergic Mechanisms of Asthma, London, United Kingdom; 4 Medical Research Council & Asthma UK Centre in Allergic Mechanisms of Asthma Protein Production Facility, London, United Kingdom

## Abstract

Immunoglobulin E (IgE) antibodies play a central role in the allergic response: interaction with FcεRI on mast cells and basophils leads to immediate hypersensitivity reactions upon allergen challenge, while interaction with CD23/FcεRII, expressed on a variety of cells, regulates IgE synthesis among other activities. The receptor-binding IgE-Fc region has recently been found to display remarkable flexibility, from acutely bent to extended conformations, with allosteric communication between the distant FcεRI and CD23 binding sites. We report the structure of an anti-IgE antibody Fab (8D6) bound to IgE-Fc through a mixed protein-carbohydrate epitope, revealing further flexibility and a novel extended conformation with potential relevance to that of membrane-bound IgE in the B cell receptor for antigen. Unlike the earlier, clinically approved anti-IgE antibody omalizumab, 8D6 inhibits binding to FcεRI but not CD23; the structure reveals how this discrimination is achieved through both orthosteric and allosteric mechanisms, supporting therapeutic strategies that retain the benefits of CD23 binding.

## Introduction

The interactions between immunoglobulin E (IgE) and its two receptors, FcεRI and CD23 (FcεRII), play pivotal roles in allergic disease^1,2^. FcεRI is principally expressed on the surface of mast cells and basophils. Allergen mediated cross-linking of FcεRI-bound IgE on the surface of these IgE-sensitized cells triggers degranulation and release of inflammatory mediators^1,2^. CD23 is expressed in membrane-bound (mCD23) and soluble forms, the latter existing as monomeric or trimeric fragments^1,3–5^. CD23, expressed on B cells and a range of other cell types, regulates a diverse set of immunological functions, including cellular adhesion, antigen presentation, regulation of growth and differentiation of B and T cells, rescue from apoptosis, release of cytotoxic and inflammatory mediators, transcytosis of IgE-immune complexes, and regulation of IgE synthesis^1,3–5^. CD23-deficient mice or those strains carrying mutated CD23 variants show a hyper-IgE phenotype^6–8^ whereas transgenic strains that overexpress CD23 show reduced levels of IgE^9,10^. Moreover, B cells, rather than FcεRI-bearing cells, are the major cell type controlling serum IgE levels in a CD23-dependent manner^11^.

IgE-Fc, the region of the antibody responsible for effector functions, comprises two identical chains, each composed of three immunoglobulin-like domains: Cε2, Cε3 and Cε4. IgE, and IgE-Fc, adopt a compact, bent structure in solution^12–18^, and the crystal structure of IgE-Fc revealed an acutely bent conformation, in which the (Cε2)_2_ domain pair folds back against the Fcε3-4 region, with an angle of 62° between the local two-fold axes of the (Cε2)_2_ domain pair and Cε4 domains^19,20^. Crystal structures of unliganded and receptor-bound forms of IgE-Fc, and the Fcε3-4 region, reveal the Cε3 domains to adopt a range of “open” and “closed” orientations relative to the Cε4 domain pair^19–29^. The interaction between IgE-Fc and FcεRIα involves an “opening” of the Cε3 domains, which engage FcεRI at two distinct sub-sites on the Cε2-proximal region of each Cε3 domain^19,25^, and IgE-Fc becomes even more acutely bent (54°) in the receptor-bound complex^12,19^. CD23 engages IgE-Fc at a region of the Cε3 domain distal to the FcεRIα binding site, and near the interface with the Cε4 domain^22,23,28,29^. Crystal structures of IgE-Fc and the Fcε3-4 region in complex with CD23 reveal this receptor to engage a range of “closed” Cε3 domain conformations^22,23,28,29^. The open and closed Cε3 domain conformations involved in FcεRIα and CD23 interactions, respectively, preclude simultaneous engagement of both receptors by IgE-Fc; binding of FcεRIα and CD23 are thus regulated in an allosteric manner^22,23,29,30^.

Unexpectedly, IgE-Fc was recently observed to undergo a large-scale conformational change^24^. An anti-IgE-Fc Fab (aεFab) captured IgE-Fc in an extended conformation, and the crystal structure of the aεFab/IgE-Fc complex revealed a fully extended, linear IgE-Fc molecule, in which the local two-fold axes of the Cε2, Cε3 and Cε4 domain pairs were coincident, and the (Cε2)_2_ domain pair no longer contacted the Fcε3-4 region^24^. This extreme conformational flexibility is suggested to underpin the different biological functions of IgE, with acutely bent FcεRI-bound IgE positioning the Fabs in an appropriate orientation for cross-linking by allergen, and the fully extended molecule, in the membrane-bound form (mIgE) as part of the B-cell receptor for antigen, extending the Fabs away from the membrane, to facilitate antigen capture^24^.

The interaction between IgE and FcεRI is a long-standing target in the treatment of allergic disease^2^. The therapeutic monoclonal anti-IgE antibody omalizumab (Xolair^®^, Novartis) is approved for the treatment of moderate-to-severe persistent allergic asthma and chronic idiopathic urticaria^31,32^. Omalizumab prevents IgE from engaging both FcεRI and CD23, decreases serum IgE levels *in vivo* by up to 95% and down-regulates surface expression of FcεRI on basophils^31,33–35^. The structural basis for the mechanism of action of omalizumab has recently been elucidated^36^; omalizumab inhibits the binding of IgE to FcεRI allosterically, as antibody binding causes the Cε3 domains to adopt a conformation that is too open to permit simultaneous engagement of both FcεRI sub-sites on IgE-Fc^36^, while the binding of IgE to CD23 is inhibited orthosterically^36,37^. Another anti-IgE antibody, MEDI4212, also inhibits the interaction between IgE-Fc and its two receptors^21^; binding to FcεRI is inhibited due to steric overlap, while binding to CD23 is inhibited as the Cε3 domains are “locked” in an open conformation^21^.

A large body of literature supports roles for CD23 both as an essential regulatory molecule in normal immune responses, and as a marker of, and mediator in, the pathophysiology of a number of human disorders. A primatized anti-CD23 antibody, lumiliximab/IDEC-152, showed modest clinical efficacy in early clinical trials for allergic asthma, where it reduced circulating IgE levels by about 40% during the period of active treatment^38,39^, but was not taken forward into late clinical trials. Indeed, there may be advantages to a therapeutic strategy that combines IgE neutralization and modulation of IgE production simultaneously.

8D6 is a monoclonal anti-IgE antibody that, like omalizumab and MEDI4212, inhibits the interaction between IgE and FcεRI^33^, thus preventing mast cell degranulation. However, 8D6, and 8D6/IgE-immune complexes, are able to engage IgE that is already bound to mCD23 on B cells^33^. An antibody with the binding characteristics of 8D6 could thus exploit the ability of CD23 to down-regulate IgE production, such as cross-linking of mCD23 by IgE-immune complexes, which inhibits B-cell proliferation and IgE synthesis^40,41^.

Here we report the 3.7 Å resolution crystal structure of IgE-Fc in complex with two 8D6 Fab fragments. IgE-Fc adopts a nearly linear conformation, exposing both faces of the Fcε3-4 region, to which the Fabs bind through a mixed protein-carbohydrate epitope. This extended IgE-Fc conformation is more compact than the conformation captured by aεFab^24^, with the (Cε2)_2_ domain pair instead contacting the Cε3 domains. The more compact IgE-Fc molecule demonstrates that not only is IgE dynamic, with the ability to flex from an acutely bent to an extended structure^24^, but the fully extended molecule itself is also conformationally diverse.

The structure of the 8D6 Fab/IgE-Fc complex reveals that FcεRI binding is inhibited both allosterically and orthosterically. By contrast, the CD23 binding site is accessible, and solution studies demonstrate that the affinity of IgE-Fc for CD23 is not substantially altered when in complex with 8D6. We thus reveal the structural basis by which IgE-Fc binding to its two principal receptors can be selectively modulated, offering insights into an alternative therapeutic approach to the treatment of allergic disease.

## Results

### Affinity of the 8D6 Fab for IgE-Fc

We performed SPR-based molecular interaction studies to measure binding stoichiometry, kinetics and affinities for the interaction of the 8D6 Fab with IgE-Fc. The 8D6 Fab was covalently coupled to an SPR sensor chip using a standard amine coupling protocol. IgE-Fc, over a range of concentrations, was flowed over the 8D6 surface, and association and dissociation rate constants were extracted from the binding curves (Supplementary Fig. S1). At the highest concentrations tested there was evidence for two distinct binding events, but at low and intermediate concentrations the interaction is well described as having a K_D_ of about 20 pM, with k_on_ = 5 × 10^6^ M^−1^ s^−1^ and k_off_ = 8 × 10^−5^ s^−1^, where the slow dissociation rate is the main contributor to the high affinity interaction.

The homodimeric character of IgE commonly results in two separate epitopes for anti-IgE antibodies and the asymmetrical structure of IgE-Fc sometimes results in two different affinities for the two epitopes^24,36^. To identify and characterize the binding of a second 8D6 Fab, we captured IgE-Fc on an 8D6 Fab sensor surface, and then measured the binding of a second 8D6 Fab to the IgE-Fc/8D6 Fab complex (Supplementary Fig. S1). Although slightly weaker than the first binding site, the second 8D6 Fab interaction is still high affinity, with a K_D_ of about 60 pM (k_on_ = 1 × 10^6^ M^−1^ s^−1^, k_off_ = 6 × 10^−5^ s^−1^). These SPR-based interaction analyses confirmed the expected 2:1 stoichiometry of 8D6 for IgE, and showed that both sites have subnanomolar binding affinities, which are higher than those for the clinically approved omalizumab^36^.

### Overall structure of the 8D6 Fab/IgE-Fc complex

**Figure 1:**
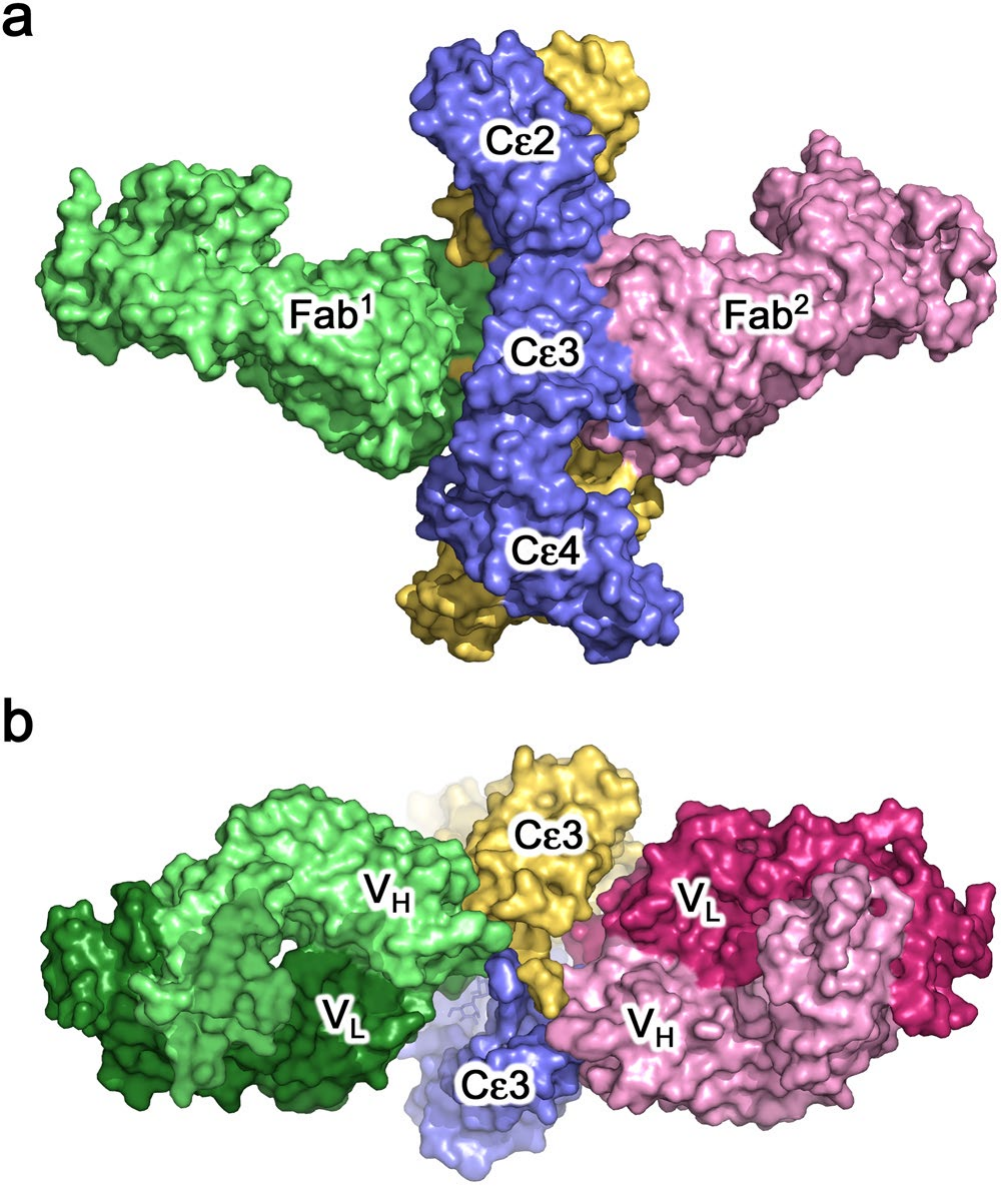
Overall structure. (**a**) Two 8D6 Fabs (light green and light pink) bind to IgE-Fc (blue and yellow) in the 8D6Fab/IgE-Fc complex, which adopts an extended conformation. (**b**) 8D6 binds the exposed face of the Fcε3-4 region. Each 8D6 Fab binds across the Cε3 domains, and forms a minor interface with one Cε2 domain [shown in a], but does not contact the Cε4 domains. The 8D6 heavy chain (light green and light pink) contributes the largest area of interaction. For clarity, the (Cε2)_2_ domain pair is not shown.

We solved the crystal structure of the 8D6 Fab/IgE-Fc complex, in which two 8D6 Fab fragments are bound to IgE-Fc, at 3.7 Å resolution. IgE-Fc adopts a fully extended conformation, exposing both faces of the Fcε3-4 region, to which the 8D6 Fabs bind (Fig. 1a,b, Supplementary Movie S1). Each 8D6 Fab binds across both Cε3 domains, and forms a minor interface with one Cε2 domain, but there is no contact with the Cε4 domains.

### The extended IgE-Fc conformation is more compact in the 8D6 Fab/IgE-Fc complex

**Figure 2:**
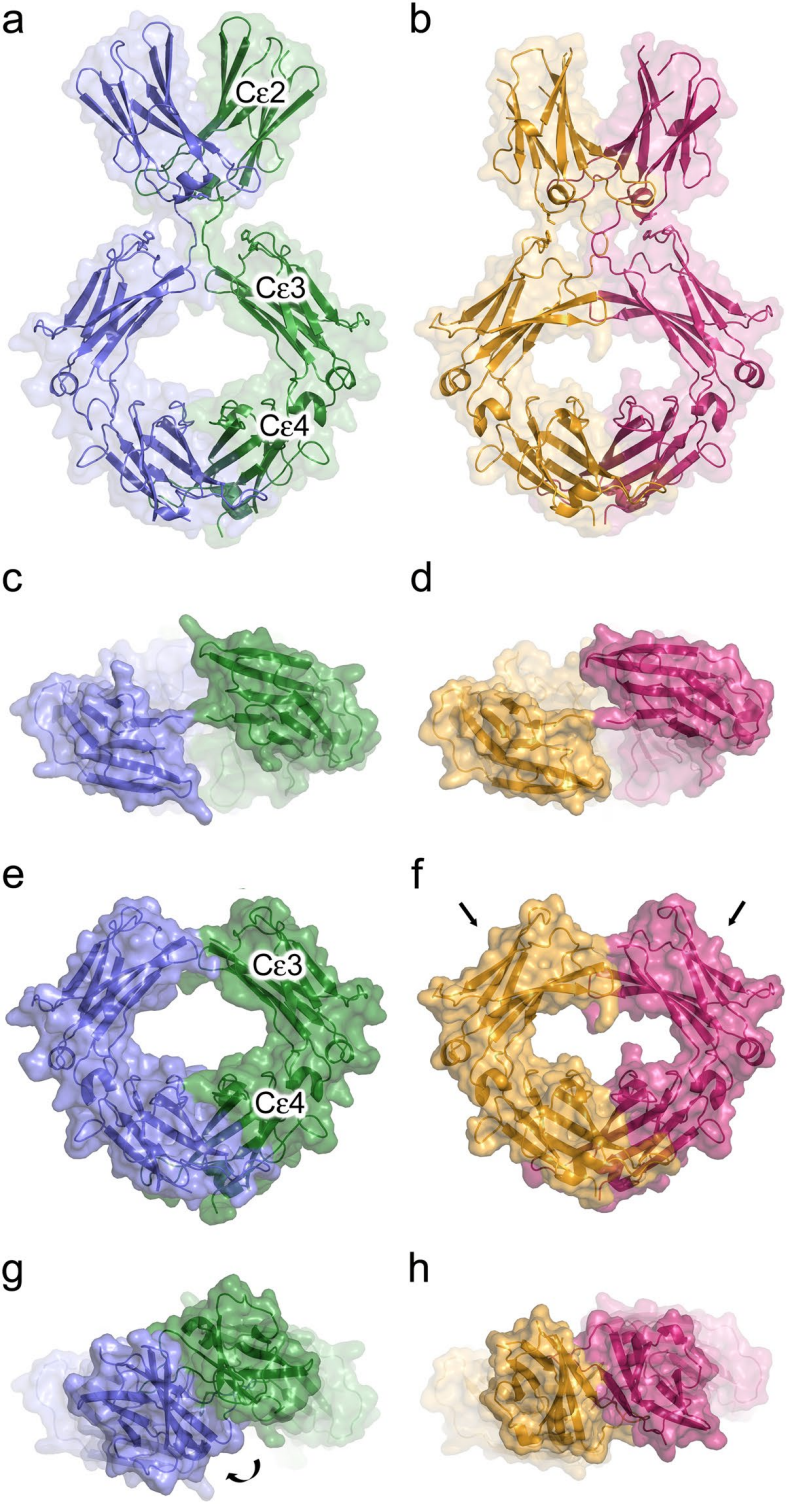
Fully extended forms of IgE-Fc. (**a**) Overall structure of IgE-Fc in the aεFab/IgE-Fc complex^24^. IgE-Fc chains A and B are coloured blue and green, respectively. (**b**) Overall structure of IgE-Fc in the 8D6 Fab/IgE-Fc complex. IgE-Fc chains A and B are coloured yellow and pink, respectively. (**c**) “Top” view of the Cε3 domains in the aεFab/IgE-Fc complex^24^. (**d**) “Top” view of the Cε3 domains in the 8D6 Fab/IgE-Fc complex. (**e**) Fcε3-4 domains in the aεFab/IgE-Fc complex. (**f**) Fcε3-4 domains in the 8D6 Fab/IgE-Fc complex. The arrows indicate that the domains adopt a more closed conformation. (**g**) (Cε2)_2_ domain pair position in the aεFab/IgE-Fc complex^24^. The curved arrow indicates that the (Cε2)_2_ domain pair rotates to generate the position observed in the 8D6 Fab/IgE-Fc complex, shown in h. (**h**) (Cε2)_2_ domain pair position in the 8D6 Fab/IgE-Fc complex.

The two-fold axes of the IgE-Fc Cε2, Cε3 and Cε4 domain pairs are coincident in the extended IgE-Fc molecule within the 8D6 Fab/IgE-Fc complex, resembling those in the fully extended conformation captured by aεFab^24^ (Fig. 2). However, further comparison of these complexes reveals substantial differences in the overall structure of IgE-Fc. In the aεFab/IgE-Fc complex, the (Cε2)_2_ domain pair is positioned too far away from the Cε3 domains to form any significant contacts. By contrast, in the 8D6 Fab/IgE-Fc complex, the Cε2 domain contacts the Cε3 domain from the same chain (Fig. 2a,b, Supplementary Fig. S2). Furthermore, the overall position of the (Cε2)_2_ domain pair relative to the Cε3 and Cε4 domains differs in the two complexes by a relative rotation of ~30° about the local two-fold axis (Fig. 2g,h). A spiral motion, resembling a corkscrew, is required to overlay the (Cε2)_2_ domain pair from the two structures, in which the (Cε2)_2_ domain pair and Cε2-Cε3 domain linker move as a rigid unit relative to the Cε3 domains (Movie S2). The overall length of the fully extended IgE-Fc molecule is ~3 Å shorter in the 8D6 Fab/IgE-Fc complex, which is thus more compact (Fig. 2a,b).

As the two IgE-Fc chains are not perfectly symmetrical in the 8D6 Fab/IgE-Fc complex, the interface between the Cε2 and Cε3 domains differs in each chain. In chain A, Leu305, Ser306 and Asp307 from the Cε2 domain form a 70 Å^2^ interface with Cε3 domain FG loop residues Pro423, His424 and Pro426, with Ser306, Asp307 and His424 contributing the largest contact area (Supplementary Fig. S2). The same residues contact one another in chain B, but form a larger interface of 113 Å^2^, with a more substantial contribution from Pro426.

In IgE-Fc and Fcε3-4 crystal structures, the Cε3 domains adopt a range of positions relative to the Cε4 domains, from “closed” to “open”^19–29,36^. The Cε3 domains adopt a more “closed” conformation in the 8D6 Fab/IgE-Fc complex compared with their position in the aεFab/IgE-Fc complex^24^ (Fig. 2c–f, Supplementary Fig. S3); this closed conformation is comparable with the range of closed conformations observed in CD23/Fcε3-4 complex crystal structures^22,23,28,29^, and is consistent with the ability of 8D6-bound IgE to bind CD23^33^.

### Cε3 domain flexibility

In addition to the variability observed for the relative domain orientations, the Cε3 domain has also been shown to possess significant intra-domain mobility; indeed the isolated Cε3 domain has molten-globule-like characteristics^42,43^. In the interaction between IgE-Fc and FcεRI, the Cε3 domain BC, C’E and FG loops play a critical role^19,25^. A comparison of the Cε3 domains in IgE-Fc and Fcε3-4 crystal structures^19–29,36,37,44,45^ reveals that the BC loop (residues 363 to 370) adopts a variety of conformations (Supplementary Fig. S4), or is partially disordered. The ordered conformations are such that the positions of certain side chains, such as Lys367, are significantly altered in different structures. Lys367 is surface exposed in the BC loop conformations found in free IgE-Fc^19,20^ and Fcε3-4^26,27^, FcεRIα-bound IgE-Fc and Fcε3-4^19,25^, and CD23-bound IgE-Fc and Fcε3-4^22,23,28,29^, and its side chain extends away from the Cε3 domain. In the aεFab/IgE-Fc complex^24^, the Lys367 side chain rests across the β-sandwich in a groove created by the Glu389, Lys391 and Leu397 side chains, and lies at one edge of the interface between IgE-Fc and the Fab. By contrast, Lys367 in the 8D6 Fab/IgE-Fc complex is wedged between the two sheets of the Cε3 β-sandwich, partially enclosed in a pocket created by Leu363, Ala364, Val370 (BC loop), His422, His424 and Leu425 (FG loop) (Supplementary Fig. S4). This conformation, which has not been observed in any other IgE-Fc or Fcε3-4 structure thus far, causes the C’E and FG loops to splay apart (Supplementary Fig. S4), and in chain B, brings Lys367 within contact distance of Asn332 (Cε2-Cε3 domain linker) in chain A (Supplementary Fig. S4). (Due to the asymmetry in IgE-Fc in the complex, Lys367 in chain A is positioned slightly further away from Asn332 in chain B).

In the 8D6 Fab/IgE-Fc complex, the BC loop forms part of the interface with CDRH1 and 3 from the Fab; BC loop conformations found in other structures are incompatible with the 8D6 Fab interface due to steric clashes. We cannot determine whether the Cε3 BC loop conformation found in the 8D6 Fab/IgE-Fc complex is induced or selected by the 8D6 Fab. Nevertheless, this structure provides further insights into the dynamic nature of the Cε3 domain, and suggests a potential role for the BC loop in the allosteric communication network in IgE-Fc that allows local conformational changes to contribute to global structural changes.

### 8D6 binds a mixed protein-carbohydrate epitope

**Figure 3:**
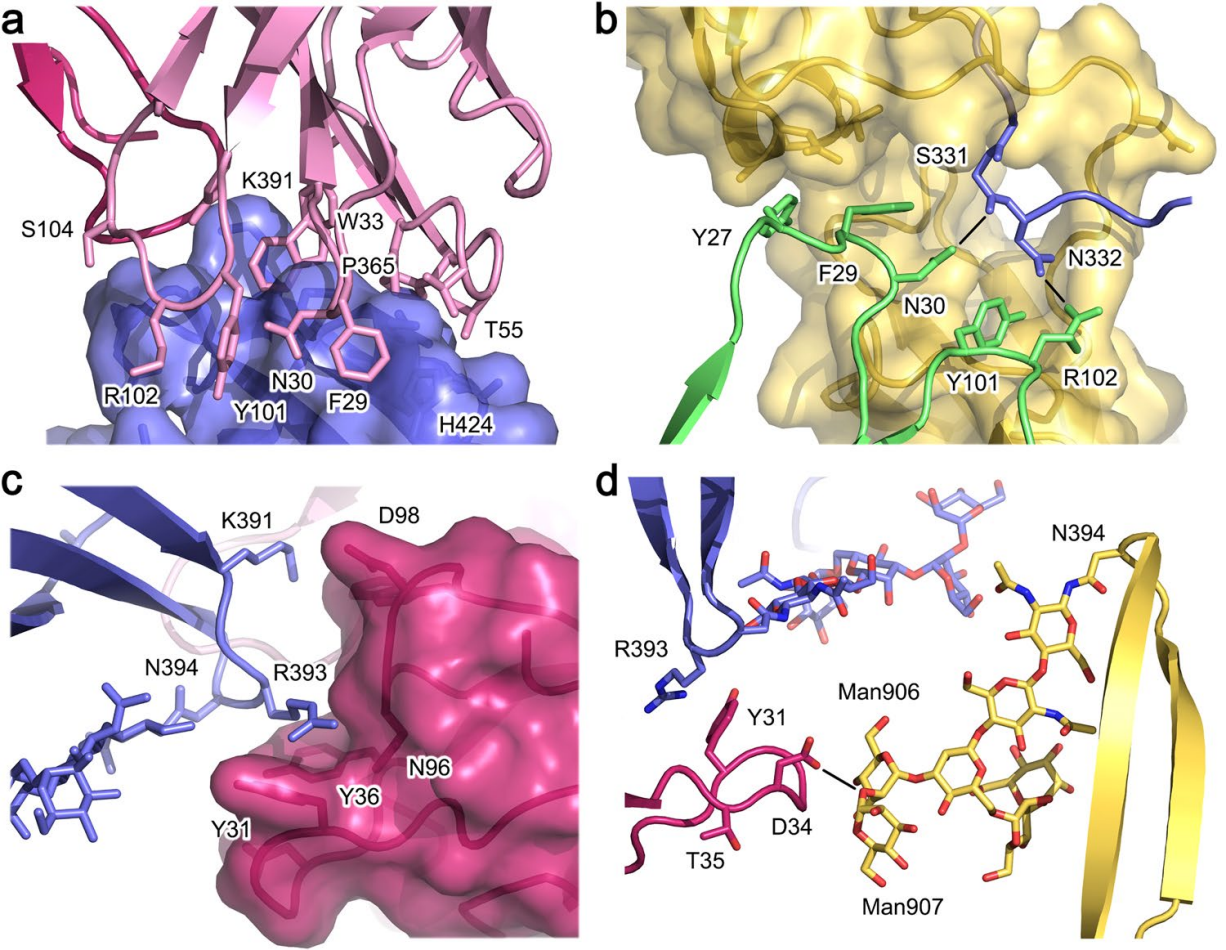
Interface between the 8D6 Fab and IgE-Fc. (**a**) The Fab heavy chain (light pink) forms a 640 Å^2^ interface with the Cε3 domain (blue). Trp33 (CDRH1) is sandwiched between the Pro365 and Lys391 (Cε3 domain) side chains. (**b**) The Fab heavy chain (green) engages the Cε2 and Cε3 domains from one IgE-Fc chain (yellow), but also contacts the Cε2-Cε3 linker from the other IgE-Fc chain (blue). Asn30 and Arg102 from the Fab are within hydrogen bonding distance of Ser331 and Asn332 (Cε2-Cε3 linker), respectively. (**c**) Tyr31, Tyr36 and Asn96 from the Fab light chain (dark pink) form a cleft around Arg393 (Cε3 domain, blue). (**d**) In a 106Å^2^ interface, Asp32, Gly33 and Asp34 from the Fab light chain (dark pink) contact two mannose residues (Man906 and Man907), and Asp34 forms a hydrogen bond with the α(1-2) glycosidic bond between Man906 and Man907.

Approximately two-thirds of the 1150 Å^2^ interface with IgE-Fc is contributed by the 8D6 heavy chain, which contacts the Cε2 and Cε3 domains from one IgE-Fc chain, and the Cε2-Cε3 domain linker from the other IgE-Fc chain. The interface between the heavy chain and Cε3 domain, covering an area of ~640 Å^2^, is similar for each Fab, and includes residues from the Cε3 domain B, C’ and E strands, and BC, C’E and FG loops. Notably, Trp33 (CDRH1) is sandwiched between the Pro365 and Lys391 (Cε3) side chains, and the interface also includes a number of hydrogen bonds (Fig. 3a). The interface with the Cε2 domain covers an area of ~140 Å^2^, in which Tyr27 and Phe29 (CDRH1) pack against Gln301, Lys302 and Leu305 (Cε2). Although the two IgE-Fc chains are not identical, similar contacts are maintained between the heavy chain and Cε2 domain at each interface; main chain atoms for residues Gly26-Phe29 (CDRH1) shift to preserve this interaction, for example, the Tyr27 Cα atom position differs by 1.5 Å (Supplementary Fig. S5). The interface with the Cε2-Cε3 linker from the other IgE-Fc chain includes a hydrogen bond between Asn30 (CDRH1) and Ser331, and both Tyr101 and Arg102 (CDRH3) side chains are within hydrogen bonding distance of Asn332 (Fig. 3b).

The 8D6 Fab light chain contributes a smaller interaction area with IgE-Fc compared with the heavy chain. In a ~180 Å^2^ interface, Tyr31 (CDRL1), Tyr36 (CDRL1) and Asn96 (CDRL3) form a cleft which accommodates the side chain of Arg393 from the same Cε3 domain contacted by the heavy chain (Fig. 3c). The Fab light chain additionally, and exclusively, contacts the high-mannose oligosaccharide moiety covalently attached to Asn394 from the other Cε3 domain. However, the nature of the light chain interaction with the carbohydrate differs for each Fab. For one Fab (92 Å^2^ interface), Asp32 and Gly33 (CDRL1) contact two mannose residues (Man906 and 907) from the α(1–3) branch, and a hydrogen bond forms between the Asp32 main chain and the α(1–3) linked mannose residue, Man906. For the other Fab (106 Å^2^ interface), Asp32, Gly33 and Asp34 (CDRL1) contact equivalent mannose residues, but a hydrogen bond forms between the Asp34 side chain and the α(1-2) glycosidic bond between Man906 and Man907 (Fig. 3d, Supplementary Fig. S6). Furthermore, due to the asymmetry in the 8D6/IgE-Fc complex, residues from the C” and D strands of this Fab also form a minor interface of ~100 Å^2^ with the Cε3 domain G strand and Cε3-Cε4 domain linker. Although glycosylation at the Asn394 site is heterogeneous, we note that the carbohydrate units engaged by 8D6 are a core structure found in all IgE molecules^46^.

### Conformational change in 8D6 upon IgE-Fc binding

To determine whether there were conformational changes in 8D6 upon binding to IgE-Fc, we solved the crystal structure of the 8D6 Fab at 2.4 Å resolution. The asymmetric unit comprises three molecules, thus providing three independent views of the uncomplexed Fab. With the exception of a 1-2 Å shift in the CDRH2 backbone, and some disorder in CDRH1 and CDRL1, the uncomplexed Fab molecules were essentially identical. However, there were significant conformational differences between unbound and complexed 8D6 Fab (Supplementary Fig. S7).

CDRH1, which is ordered in only one of the unbound 8D6 Fab structures, contacts the Cε2 domain and the Cε2-Cε3 linker in the 8D6 Fab/IgE-Fc complex. To form this interaction, substantial shifts occur in the positions of Thr28, Phe29 and Asn30 (CDRH1), but the most dramatic change occurs in the Tyr27 (CDRH1) side chain, which flips from one face of the main chain to the other to contact Gln301 and Lys302 from the Cε2 domain. Conformational flexibility in CDRH1 appears to be facilitated by the presence of two glycine residues, at positions 26 and 31, whereas in the complex, steric constraints are imposed by the Cε3 domain residues Pro365 and Ser366 (Supplementary Fig. S7).

### Inhibition of FcεRI binding by 8D6

**Figure 4:**
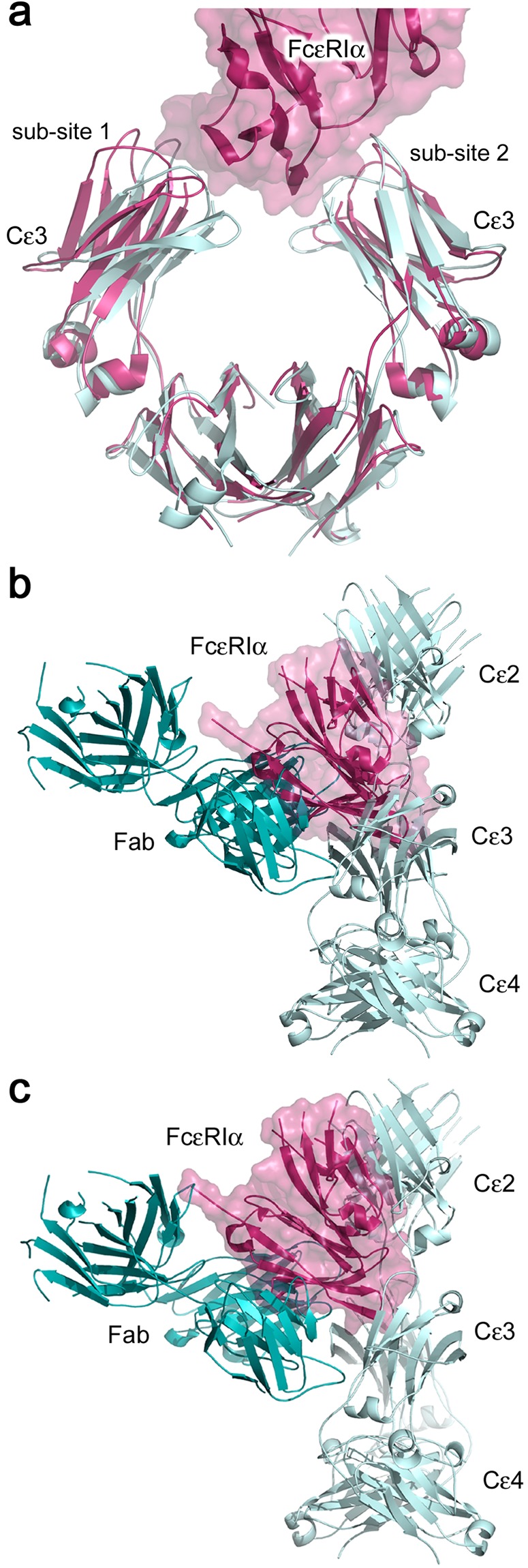
Structural basis for inhibition of the interaction between IgE-Fc and FcεRI by 8D6. (**a**) The Cε3 domains in the 8D6 Fab/IgE-Fc complex (light blue) adopt a closed conformation, precluding simultaneous engagement of both sub-sites on sFcεRIα. The 8D6 Fab/IgE-Fc complex and sFcεRIα/IgE-Fc complex (pink)^19^ structures were superposed on Cε4 domain Cα atoms. (**b**) When the 8D6 Fab/IgE-Fc and IgE-Fc/sFcεRIα complex structures are superposed on the Cε3 domain which engages FcεRIα at sub-site 1, one Fab (dark blue) and the (Cε2)_2_ domain pair (light blue) from the 8D6 Fab/IgE-Fc complex clash with sFcεRIα (pink). For clarity, IgE-Fc from the IgE-Fc/sFcεRIα complex is not shown. (**c**) When the 8D6 Fab/IgE-Fc and IgE-Fc/sFcεRIα complex structures are superposed on the Cε3 domain which engages FcεRIα at sub-site 2, one Fab (dark blue) and the (Cε2)_2_ domain pair from the 8D6 Fab/IgE-Fc complex (light blue) clash with sFcεRIα (pink). For clarity, IgE-Fc from the IgE-Fc/sFcεRIα complex is not shown.

The high affinity interaction between IgE and FcεRI (K_a_ = 10^10^ − 10^11^ M^−1^) involves an acutely bent conformation for IgE-Fc, in which the Cε3 domains adopt an open conformation, and engage the receptor through two distinct sub-sites^1,2,19^. The anti-IgE 8D6 antibody inhibits binding of IgE to FcεRI, and is also unable to engage FcεRI-bound IgE^33^. Comparison of the IgE-Fc/FcεRIα and 8D6 Fab/IgE-Fc complexes reveals that the Cε3 domains adopt a conformation that is too closed to permit simultaneous engagement at both FcεRIα sub-sites (Fig. 4a). Furthermore, comparison at each sub-site of receptor engagement^19^ reveals steric clashes between the 8D6 Fab and FcεRIα, and the (Cε2)_2_ domain pair and FcεRIα (Fig. 4b,c). Inhibition of FcεRI binding by 8D6 thus involves both allosteric and orthosteric mechanisms.

### Interaction between 8D6-bound IgE-Fc and CD23

**Figure 5:**
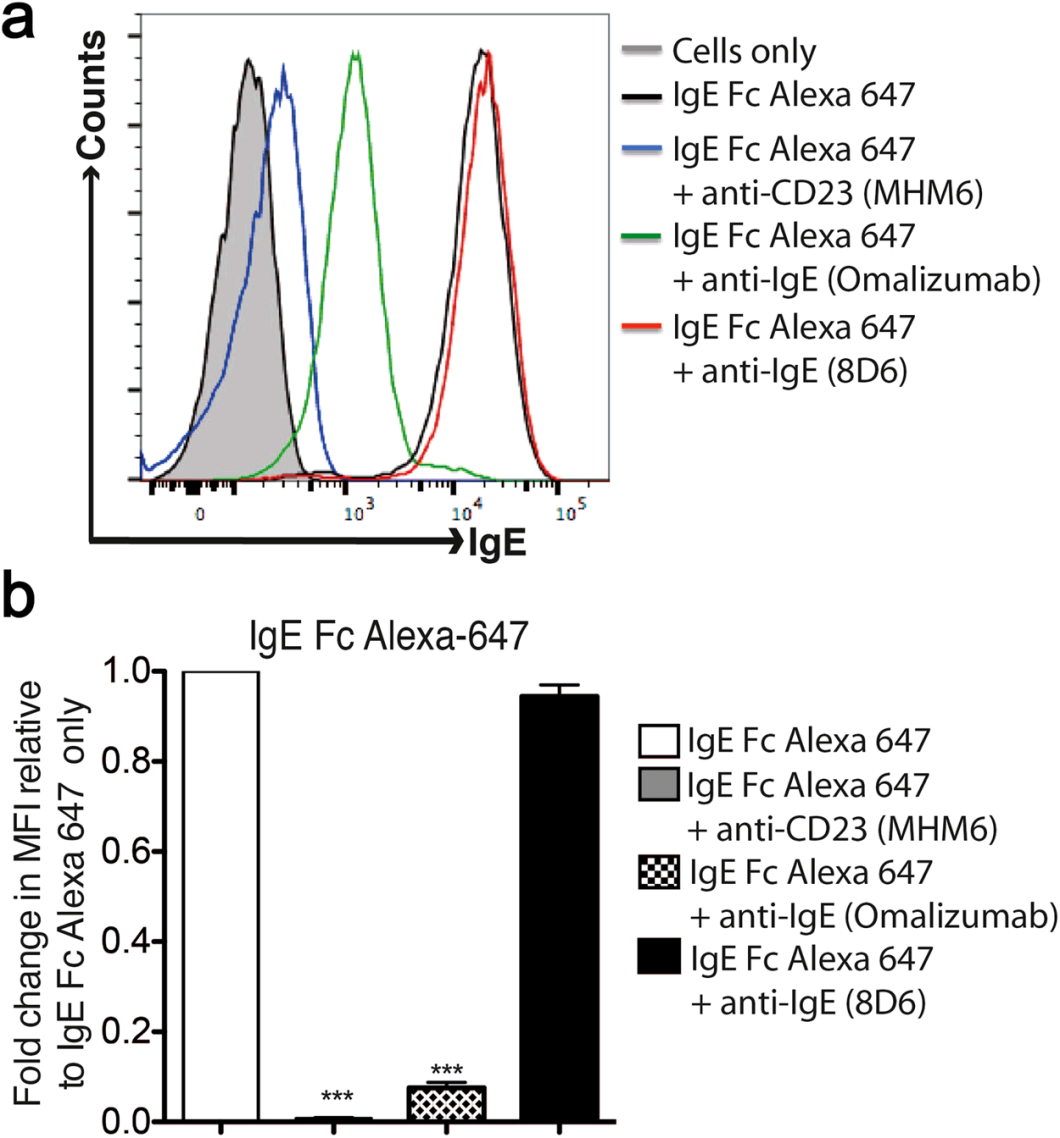
Effect of MHM6 (anti-CD23), omalizumab (anti-IgE) and 8D6 (anti-IgE) antibodies on IgE-Fc binding to membrane CD23 (mCD23) on RPMI 8866 cells. IgE-Fc Alexa 647 was added to 0.5 × 10^6^ RPMI 8866 cells with or without MHM6, omalizumab or 8D6 at a molar ratio of 1:2 for 30 min at 4 °C. Cells were then washed and acquired for flow cytometry analysis. (**a**) Histograms show the levels of IgE-Fc bound to mCD23 on RPMI 8866 cells as determined by flow cytometry. (**b**) Summary of the mean fluorescence intensity (MFI) made relative to IgE-Fc Alexa 647 alone (n = 6). Statistical analysis was performed using the One-Way ANOVA, with Bonferroni correction. A *p* value of < 0.05 was considered significant (*p < 0.05,**p < 0.01,***p < 0.001).

To further characterize the previously reported interaction between 8D6-bound IgE and CD23^33^ we investigated binding of fluorescently labeled IgE-Fc to membrane CD23 (mCD23) on RPMI 8866 cells, obviating the need for secondary IgE detection antibodies. We observed that IgE-Fc alone was able to bind mCD23, but failed to do so in the presence of MHM6 (anti-CD23) and omalizumab (anti-IgE), known inhibitors of the IgE/CD23 interaction^33,47^ (Fig. 5). In contrast, 8D6 did not inhibit IgE-Fc binding to mCD23 (Fig. 5).

**Figure 6:**
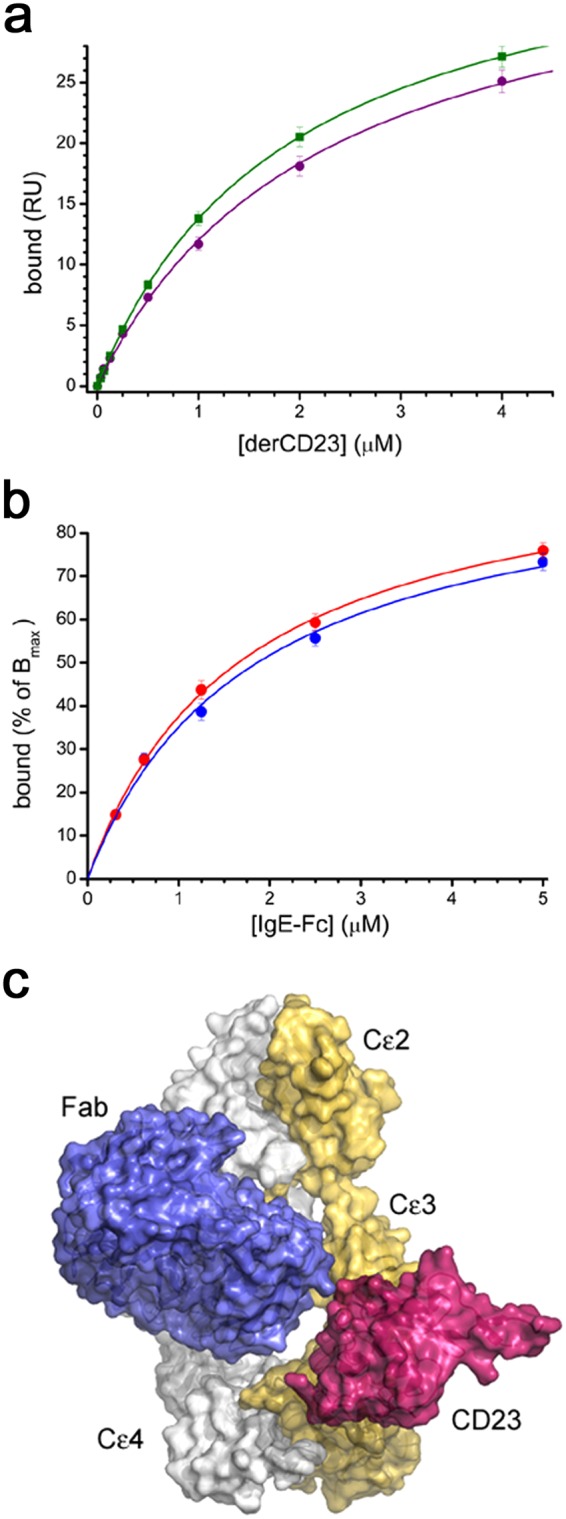
The 8D6 Fab does not prevent CD23 binding to IgE-Fc. (**a**) A comparison of the binding of derCD23 to either unbound IgE-Fc (green) or an 8D6/IgE-Fc complex (purple). The binding affinity of derCD23 to IgE-Fc captured on an SPR sensor surface via an N-terminal His-tag was 1.6 (±0.1) μM; the binding affinity of derCD23 to IgE-Fc captured using the 8D6 Fab and then saturated with 8D6, to occupy both 8D6 binding sites on IgE-Fc, was 1.7 (±0.1) μM. (**b**) The binding of IgE-Fc (red) to derCD23 immobilized on an SPR sensor surface, was compared with the binding of a 2:1 8D6 Fab/IgE-Fc complex (blue) to the same derCD23 surface. SPR signal is mass dependent, so binding was normalized according to maximal binding (B_max_) in the fitted curve. When this normalization is done, the binding curves for unbound IgE-Fc and the IgE-Fc/8D6 Fab  complex are nearly identical. (**c**) 8D6 and derCD23 bind to different sites on IgE-Fc. Comparison of the 8D6 Fab/IgE-Fc complex and a CD23/Fcε3-4 complex^22^ structure reveals that there is no steric overlap between 8D6 and CD23 in chain B of the 8D6 Fab/IgE–Fc complex. IgE-Fc chains A and B from the 8D6 Fab/IgE-Fc complex are coloured grey and yellow, respectively, the 8D6 Fab is coloured blue, and CD23 from the CD23/Fcε3-4 complex^22^, dark pink. Structures were superposed on the Cε3 domain (chain B) from the 8D6 Fab/IgE-Fc complex. For clarity, the Fcε3-4 region from the CD23/Fcε3-4 complex is not shown.

We next investigated the interaction between 8D6-bound IgE-Fc and CD23 using surface plasmon resonance. A soluble, monomeric CD23 construct, called derCD23^48^, binds to IgE-Fc captured on an 8D6 Fab surface (K_D_ = 1.7 ± 0.1 μM) with the same affinity as IgE-Fc captured via an N-terminal His-tag (K_D_ = 1.8 ± 0.2 μM (Fig. 6a). This demonstrates that the 8D6 Fab, bound in a 1:1 complex with IgE-Fc, does not inhibit the interaction between IgE-Fc and derCD23. We also wished to determine whether a 2:1 complex between the 8D6 Fab and IgE-Fc would still bind CD23. We captured IgE-Fc on an 8D6 Fab surface, bound a second 8D6 Fab to this complex at concentrations sufficient to saturate the second binding site, and then measured binding of derCD23, over a range of concentrations, to the 2:1 8D6 Fab/IgE-Fc complex. We observed that binding of derCD23 to the 2:1 8D6 Fab/IgE-Fc complex (K_D_ = 1.7 ± 0.2 μM) was similar to binding of derCD23 to unbound IgE-Fc (K_D_ = 1.7 ± 0.2 μM) (Fig. 6b). The lack of competition between 8D6 and CD23 is consistent with their distal epitopes (Fig. 6c).

CD23 binds to IgE when the Cε3 domains adopt a closed conformation; CD23 engages the Cε3 domain distal to the FcεRIα binding site, and the interface extends to include residues from the Cε4 domain^22,23,28,29^. Omalizumab inhibits the interaction between IgE and CD23 orthosterically^36,37^, while MEDI4212 (anti-IgE) “locks” the Cε3 domains in an open conformation, precluding the interaction with CD23^21^ (Supplementary Fig. S8). The closed Cε3 domain conformation in the 8D6 Fab/IgE-Fc complex is similar to the range of closed conformations observed in the eighteen independent views of the interface between CD23 and IgE-Fc and Fcε3–4^22,23,28,29^, and the CD23 binding site itself is also comparable. Further structure comparison revealed no steric clashes between CD23 and the 8D6 Fab when chain B from the 8D6 Fab/IgE-Fc complex structure was compared (Fig. 6c), although minor steric clashes between CD23 and 8D6 were observed for chain A. Given that the interface between 8D6 and one IgE-Fc chain, mediated by the 8D6 light chain, is almost exclusively composed of interactions with the oligosaccharide moiety covalently attached to Asn394, and that this interface is flexible (Supplementary Fig. S6), it is likely that IgE-Fc could undergo a further conformational change in solution which would render the second binding site fully accessible.

## Discussion

IgE-Fc adopts a predominantly bent conformation in solution^12–18^, and the crystal structure of IgE-Fc, alone and when in complex with FcεRI, is acutely bent^19,20^. IgE has since been discovered to be more conformationally dynamic than previously thought, after a fully extended conformation was unexpectedly captured in the crystal structure of IgE-Fc in complex with an anti-IgE Fab (aεFab)^24^, and a partially bent conformation, with markedly open Cε3 domains, was observed in the crystal structure of IgE-Fc in complex with an omalizumab-derived Fab^36^.

Here, we report the 3.7 Å resolution crystal structure of IgE-Fc in complex with the Fab fragment of the anti-IgE antibody 8D6, in which two 8D6 Fabs bind to a fully extended IgE-Fc molecule. The 8D6 Fab/IgE-Fc complex reveals a more compact form of fully extended IgE-Fc, in which the Cε3 domain contacts the Cε2 domain from the same chain, and the Cε2-Cε3 domain linker from the other chain. The 8D6 Fab/IgE-Fc complex reveals that not only is IgE-Fc flexible, adopting both acutely bent and extended conformations, but the fully extended conformation itself is also conformationally diverse. A spiral motion, resembling that of a corkscrew, in which the Cε3 domains close, and the (Cε2)_2_ domain pair and Cε2-Cε3 domain linker rotate and move closer to the Cε3 domains, transforms one fully extended form to the other. Furthermore, the unique Cε3 BC loop conformation loop conformation found in the 8D6 Fab/IgE-Fc complex suggests that local conformational changes in IgE-Fc contribute to global structural changes.

It remains to be determined whether the compact, extended form observed is induced, or selected, by 8D6, but the extended conformation previously observed in the aεFab/IgE-complex was captured by the Fab^24^. Given the potential for extreme flexibility, it is therefore possible that IgE-Fc can freely adopt this compact, extended conformation in solution.

In a recent molecular dynamics study exploring the unbending of IgE-Fc to an extended conformation, the acutely bent conformation occupied the lowest energy basin^24^, but the extended conformation captured by aεFab occupied a high-energy basin^24^. Given the proposed role for the extended form of IgE as part of the B-cell receptor for antigen, the more compact form found in the 8D6 Fab/IgE-Fc complex provides clues about how a fully extended IgE conformation might be stabilized, even transiently, on the B-cell surface, to facilitate antigen capture.

The interaction between IgE and FcεRI, a prerequisite for mast cell degranulation, is a long-standing target in the treatment of allergic disease^2^, and is inhibited by the clinically approved therapeutic antibody omalizumab^31^, and other anti-IgE antibodies such as MEDI4212^21^, aεFab^24^ and 8D6^33^; however, the mechanism of inhibition for these antibodies differs. In the complex of an omalizumab-derived Fab with IgE-Fc, the Cε3 domains adopt a markedly open conformation, even more open than that for FcεRI-bound IgE-Fc^36^, which precludes simultaneous engagement of the two FcεRI-binding sub-sites^36^, while the complex of MEDI4212 with Fcε3-4 reveals steric overlap with FcεRI^21^. In the aεFab/IgE-Fc complex, structural rearrangements in the Cε2-Cε3 domain linker, disruption of a key receptor interaction, and steric clashes between the extended IgE-Fc conformation and FcεRI, preclude receptor binding^24^. In the 8D6 Fab/IgE-Fc complex, the Cε3 domains adopt a conformation which is too closed to permit simultaneous engagement of the FcεRI-binding sub-sites, and the Cε2 domains in the extended IgE-Fc conformation would also clash with the receptor.

Unlike the clinically-approved omalizumab, 8D6 does not inhibit the interaction between IgE and CD23^33^. We have demonstrated here that 8D6-bound IgE-Fc binds to mCD23 on cells, and that the affinity of the 2:1 8D6/IgE-Fc complex for a soluble fragment of CD23 (derCD23) is similar to unbound IgE-Fc. Consistent with these observations, the crystal structure of the 2:1 8D6 Fab/IgE-Fc complex reveals that one of the CD23 binding sites on IgE-Fc is fully accessible, with only minor clashes at the second site; a minor conformational change in IgE-Fc, involving the flexible 8D6 epitope, which includes the oligosaccharide moiety, would render the second CD23 binding site accessible.

In its role as a regulator of IgE levels, engagement of mCD23 by IgE-immune complexes inhibits B-cell proliferation and IgE synthesis^3,40,41^. Inhibiting the interaction between IgE and FcεRI, but not CD23, is thus a potentially promising therapeutic approach in the treatment of allergic disease. Here, we have revealed the structural basis by which binding of IgE to these two key receptors can be selectively modulated.

## Materials and Methods

### Protein expression, purification and labeling

Human IgE-Fc used for the crystallographic study and FACS analysis, and human derCD23 used for the SPR analysis, were expressed as previously described^48,49^. Recombinant 8D6 Fab was expressed in Freestyle HEK293F cells using a previously published method^50^ with the exception that after a four hour transfection at high cell density, the cells were diluted four-fold in the presence of 3.75 mM sodium valproate, and after seven days the culture supernatant was harvested and protein purified by KappaSelect affinity chromatography. His-tagged IgE-Fc used for the SPR analysis contained the same sequence as that used for the crystallographic study (human IgE-Fc starting at residue Val224, with Cys225Ala, Asn265Gln and Asn371Gln mutations), in addition to an N-terminal tag (6xHis and Xpress tags), was expressed in Freestyle™ HEK293F cells, and purified by Ni^2+^-affinity chromatography.

The 8D6 Fab/IgE-Fc complex for the crystallographic study was purified as follows: 8D6 Fab and IgE-Fc proteins were mixed at a molar ratio of 2.5:1 at 4 °C for 16 hours and the resulting complex purified by size exclusion chromatography using a Superdex 200 column.

For the flow cytometry analysis, IgE-Fc was concentrated to 50 µM, and dialysed overnight at 4 °C against a buffer of 100 mM sodium bicarbonate and 50 µM NaCl, pH 9. A 2.5 molar excess of Alexa Fluor® 647 succinimidyl ester (Life Technologies) was added to IgE-Fc, gently agitated for 3 hours, and then dialysed against PBS overnight. Absorbance of the sample at 280 nm and 647 nm was used to determine the labeling efficiency.

### Surface plasmon resonance analysis

Experiments were performed on a Biacore T200 instrument (GE Healthcare). Specific binding surfaces were prepared by coupling 8D6 Fab to a CM5 sensor chip, using a standard amine coupling protocol (GE Healthcare). For measuring the affinity of the first binding site of 8D6 Fab to IgE-Fc, His-tagged IgE-Fc, at concentrations ranging from 100 nM to 190 pM, prepared by a 2-fold serial dilution in HBS-P buffer (10 mM HEPES, pH 7.4, 150 mM NaCl, 0.005% surfactant p20), was injected at 30 μL/min, with a 2 min association phase followed by a 12 min dissociation phase. For the sandwich binding experiments, approximately 100 resonance units of His-tagged IgE-Fc was captured on an 8D6 Fab surface; after a 2 min stabilization period, 8D6 Fab was injected at 30 μL/min with a 2 min association phase followed by a 12 min dissociation phase. The sensor surface was regenerated by two 30 sec injections of 0.2 M glycine-HCl, pH 2.3.

For measuring the binding of derCD23 to IgE-Fc or 8D6 Fab-bound IgE-Fc, all experiments were performed in HBS-P buffer with 5 mM CaCl_2_. DerCD23 binding to (1) N-terminally His-tagged IgE-Fc captured using an anti-His-tag antibody (Biacore His Capture Kit, GE Healthcare), (2) IgE-Fc captured on an 8D6 Fab surface, resulting in derCD23 binding to a 1:1 8D6 Fab/IgE-Fc complex, and (3) IgE-Fc captured on an 8D6 Fab surface, and then bound to a second 8D6 Fab by injecting a saturating concentration (100 nM) of 8D6 Fab, resulting in derCD23 binding to a 2:1 8D6 Fab/IgE-Fc complex. DerCD23 solutions over a range of concentrations (10, 5, 2.5, 1.25, 0.625, 0.312, 0.156 and 0 μM) were injected at 20 μL/min with a 60 sec association phase, followed by a 90 sec dissociation phase. All measurements were done in duplicate, using titration series of “low-to-high” analyte concentration followed by a “high-to-low” series^51^ at 25 °C. In all cases, standard double referencing data subtraction methods were used^51^ and kinetic fits were performed using Origin software (OriginLab).

### Flow cytometry analysis of IgE binding to membrane CD23 on RPMI 8866 cells

To examine the effect of 8D6 on IgE binding to membrane CD23, 0.5 × 10^6^ cells from a CD23 expressing cell line (RPMI 8866), maintained as described previously^52^, were incubated with 5 μg/mL of IgE-Fc Alexa-647 with or without 10 μg/mL 8D6 (anti-IgE). MHM6 (anti-CD23) and omalizumab (anti-IgE), two known inhibitors of IgE binding to CD23^47^, were used as controls. After 30 minutes of incubation on ice, cells were washed by centrifugation at 300 × *g* for 5 mins at 4 °C, collected using a BD FACSCanto and analysed using FlowJo software (Tree Star Inc).

### Crystallization

For crystallization trials, the 8D6 Fab was buffer-exchanged into 20 mM Tris-HCl pH 8.6, and concentrated to an OD_280_ of 11. 8D6 Fab crystals were grown by sitting drop vapour diffusion at 18 °C in MRC 96 well plates. The reservoir contained 50 µL of 0.1 M ADA (N-(2-acetamido) iminodiacetic acid) pH 6.5 and 12% (w/v) PEG 4000. The drop contained 250 nL and 400 nL of protein solution and reservoir, respectively. Crystals were cryoprotected in 0.1 M Tris-HCl pH 7.0, 20% (w/v) PEG 3350, 0.2 M (NH_4_)_2_SO_4_ and 18% (v/v) ethylene glycol before flash-cooling in liquid nitrogen. Purified 8D6 Fab/IgE-Fc complex was buffer-exchanged into 100 mM Tris-HCl pH 7.4 and 50 mM NaCl, and concentrated to an OD_280_ of 16.4. Crystals of the 8D6 Fab/IgE-Fc complex were also grown by sitting-drop vapour diffusion. The reservoir contained 75 µL of 0.1 M Tris-HCl pH 8.0 and 45% (v/v) pentaerythritol propoxylate (5/4PO/OH). The drop contained 100 nL and 50 nL of protein solution and reservoir, respectively. Crystals were flash-cooled in liquid nitrogen using mother liquor as a cryoprotectant.

### X-ray data collection, structure determination and refinement

Data for the 8D6 Fab were collected at beamline I04 at the Diamond Light Source (Harwell, UK). Intensities were integrated with XDS using the xia2 package^53,54^, and further processed to 2.4 Å resolution with programs from the CCP4 suite^55,56^. The structure was solved in space group *P*4_3_, with three Fab molecules in the asymmetric unit. The structure was solved by molecular replacement with PHASER^57^ using protein atoms from PDB entry 1I7Z^58^ as a search model. Data for the 8D6 Fab/IgE-Fc complex were collected at beamline I04-1 at the Diamond Light Source. Intensities were integrated with XDS^53^ and further processed to 3.7 Å resolution with programs from the CCP4 suite^55,56^. The structure was solved in space group *P*2_1_2_1_2_1_, using a combination of molecular replacement with PHASER^57^ and manual domain placement, using a partially refined 8D6 Fab structure and protein atoms from PDB entry 2WQR as search models^19^, and the asymmetric unit contained one 2:1 8D6 Fab/IgE-Fc complex. Both structures were refined with PHENIX^59^. Manual model building was performed with Coot^60^. Overall structure quality was assessed with MolProbity^61^ and POLYGON^62^ within PHENIX. Data processing statistics are summarized in Table 1. A representative electron density map for each structure is shown in Supplementary Fig. S9. Interfaces were analysed with PISA^63^, figures were produced with PyMOL^64^, and movies were generated using Chimera^65^, PyMOL^64^ and the eMovie plugin^66^ for PyMOL.

**Table 1:** Data processing and refinement statistics.

	8D6 Fab	8D6Fab/IgE-Fc complex
***Data processing***		
Space group	*P* 4_3_	*P* 2_1_ 2_1_ 2_1_
**Unit cell dimensions**		
*a*, *b*, *c* (Å)	157.96, 157.96, 77.50	119.48, 119.60, 132.99
Resolution (Å)	70.64–2.40 (2.45–2.40)^a^	88.93–3.70 (4.05–3.70)^a^
No. of unique reflections	74 039 (4 617)^a^	20 732 (4 698)^a^
Completeness (%)	98.8 (99.9)^a^	98.8 (95.3)^a^
Multiplicity	6.2 (6.3)^a^	6.6 (6.8)^a^
Mean ((*I*)/σ(*I*))	10.5 (1.9)^a^	6.8 (2.8)^a^
R_merge_ (%)	12.3 (147.6)^a^	22.4 (69.1)^a^
R_pim_ (%)	4.9 (58.1)^a^	9.4 (28.4)^a^
CC_1/2_	0.996 (0.643)	0.989 (0.819)
***Refinement***		
*R*_work_/*R*_free_ (%)^b^	18.13/21.21	24.73/28.82
**RMSD**		
Bond lengths (Å)	0.003	0.002
Bond angles (°)	0.643	0.510
Coordinate error (Å)	0.32	0.49
**No. of atoms**		
Protein^c^	9 152	10 230
Carbohydrate	0	155
Solvent	249	0
Other^d^	59	0
**Ramachandran plot**		
Favoured (%)	96.59	96.21
Allowed (%)	3.33	3.65

^a^Numbers in parentheses are for the highest resolution shell.

^b^R_free_ set comprises 5% of reflections.

^c^Includes alternative conformations.

^d^Ethylene glycol, polyethylene glycol, sulphate.

### Data availability

Coordinates and structure factors have been deposited at the Protein Data Bank with accession codes 6EYN (8D6 Fab) and 6EYO (8D6 Fab/IgE-Fc complex).

## Electronic supplementary material

Supplementary Information

Supplementary Movie S1

Supplementary Movie S2
