# Supplementary material for: Structural basis for selective inhibition of immunoglobulin E-receptor interactions by an anti-IgE antibody
Source: Sci Rep. 2018 Aug 1;8:11548. doi: 10.1038/s41598-018-29664-4 (PMC6070508; doi:10.1038/s41598-018-29664-4)
Supplement: Supplementary file 1 — Supplementary Information [file 41598_2018_29664_MOESM1_ESM.pdf]

Supplementary Information

Structural basis for selective inhibition of Immunoglobulin E-receptor interactions by an anti-IgE antibody

Jiun-Bo Chen[1,2], Faruk Ramadani[2,3], Marie O. Y. Pang[2,3], Rebecca L. Beavil[2,3,4], Mary D. Holdom[2,3], Alkistis N. Mitropoulou[2,3], Andrew J. Beavil[2,3], Hannah J. Gould[2,3], Tse Wen Chang[1], Brian J. Sutton[2,3*], James M. McDonnell[2,3]* and Anna M. Davies[2,3]*

Author Affiliations

[1] Genomics Research Center, Academia Sinica, Taipei 115, Taiwan.

[2] King's College London, Randall Centre for Cell and Molecular Biophysics, London SE1 1UL, United Kingdom.

[3] Medical Research Council & Asthma UK Centre in Allergic Mechanisms of Asthma, London, United Kingdom.

[4] Medical Research Council & Asthma UK Centre in Allergic Mechanisms of Asthma Protein Production Facility, London, United Kingdom.

* E-mail: anna.davies@kcl.ac.uk
* E-mail: james.mcdonnell@kcl.ac.uk
* E-mail: brian.sutton@kcl.ac.uk

Contents

1. Supplementary Figure S1
2. Supplementary Figure S2
3. Supplementary Figure S3
4. Supplementary Figure S4
5. Supplementary Figure S5
6. Supplementary Figure S6
7. Supplementary Figure S7
8. Supplementary Figure S8
9. Supplementary Figure S9
10. Supplementary Movie Captions
11. Supplementary References

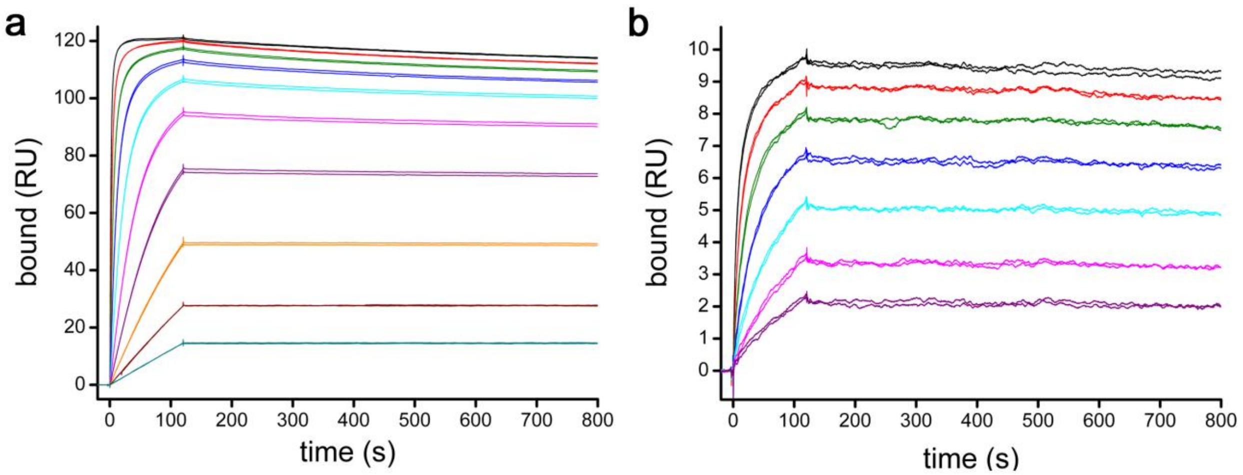

Supplementary Figure S1. 8D6 Fab binds to IgE-Fc with low picomolar affinity and a 2:1 stoichiometry. (A) Direct binding was measured for IgE-Fc to immobilized 8D6 Fab. The Fab was covalently immobilized at low density using an amine coupling kit (GE Healthcare) and IgE-Fc was flowed over this surface at a variety of concentrations, using a two-fold dilution series with a highest concentration of 100 nM. (B) The binding of the second 8D6 Fab binding site was characterized using an SPR sandwich binding experiment. IgE-Fc was first captured on an 8D6 Fab surface, then a second 8D6 Fab molecule was added to the IgE-Fc/8D6 Fab complex, in a two-fold dilution series starting at a concentration of 100 nM. For all binding experiments, all concentrations were run in duplicate and standard double referencing methods were employed [1].

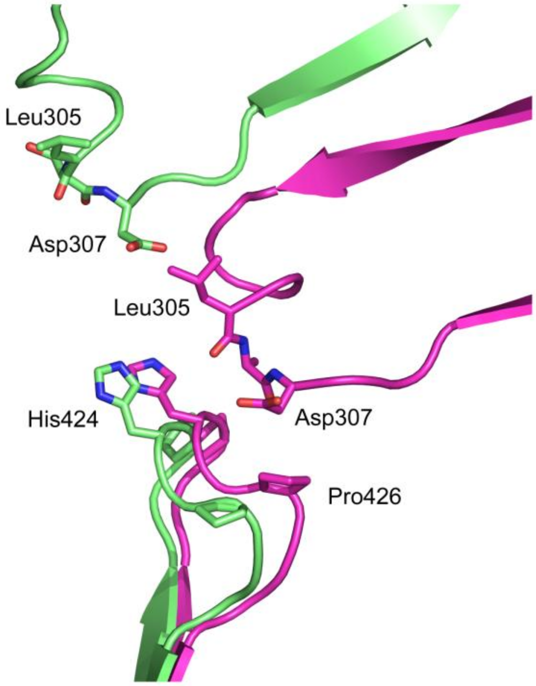

Supplementary Figure S2. Contact between the Cε2 and Cε3 domains. In the 8D6 Fab/IgE-Fc complex (pink), residues Leu305-Asp307 from the Cε2 domain contact residues Pro423, His424 and Pro426 from the Cε3 domain. In the aεFab complex (green) [2], the position of the Cε2 domain precludes an interaction with the Cε3 domain. The figure was generated after superposing the Cε3 domains from chain B of the 8D6 Fab/IgE-Fc and aεFab/IgE-Fc complexes.

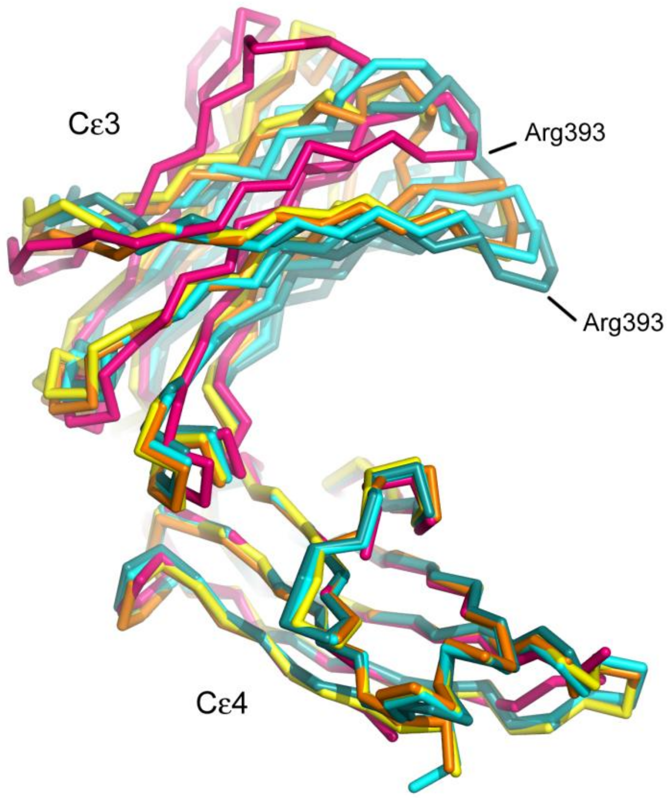

Supplementary Figure S3. Cε3 domain conformation. The Cε3 domains adopt a closed conformation in the 8D6 Fab/IgE-Fc complex (chain A, orange; chain B, yellow), akin to the closed conformation observed in the CD23/Fcε3-4 complex (PDB 4EZM; chain B, cyan; chain D, teal) [3]. In contrast, the Cε3 domain adopts an open conformation in the sFcεRIα/IgE-Fc complex (PDB 2Y7Q; chain B, pink) [4]. Structures were superposed on Cε4 domain Cα atoms.

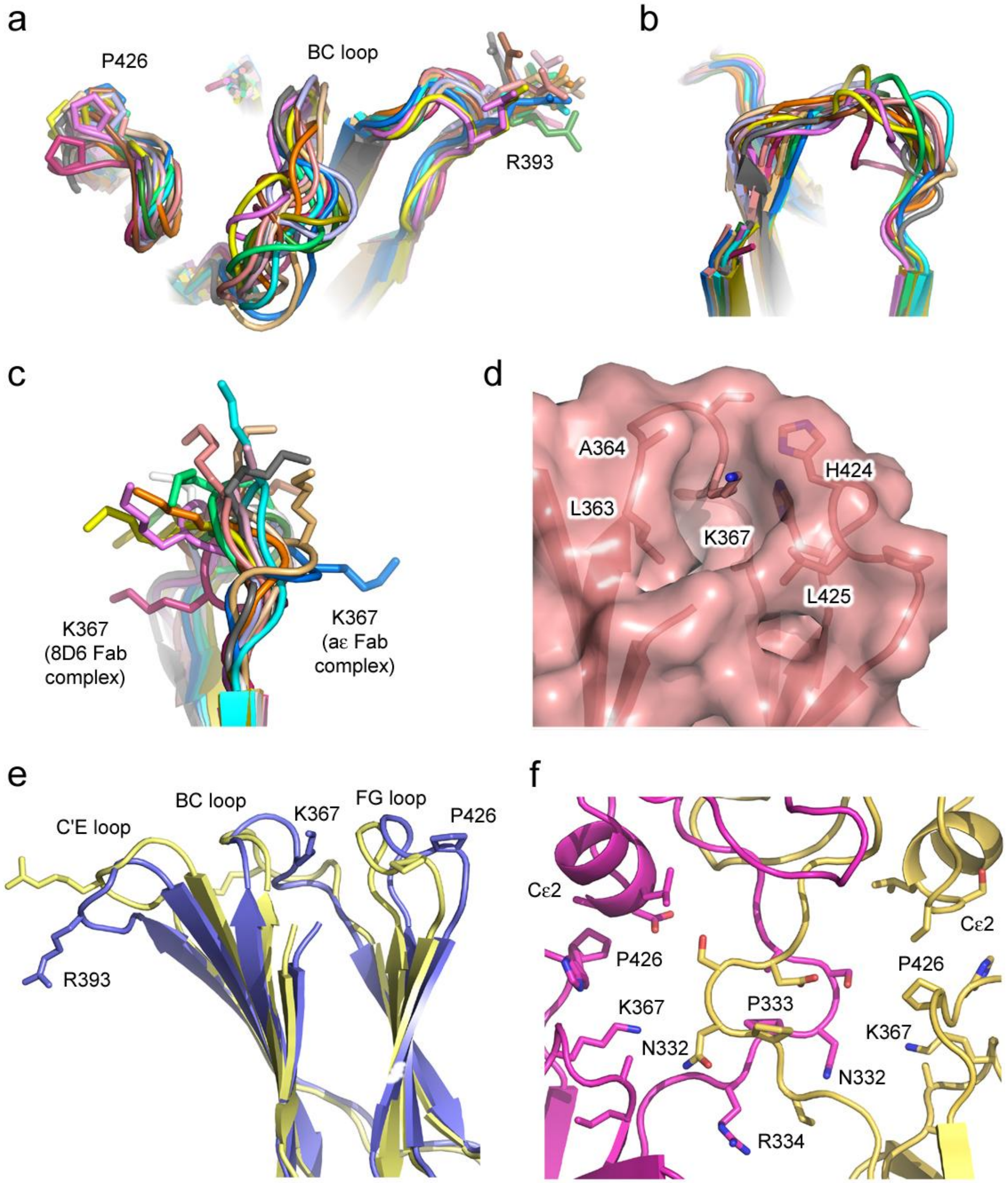

Supplementary Figure S4. The Cε3 domain BC loop. (A) Top view of the Cε3 domain, showing the range of conformations adopted by the BC loop in different structures. (B) Side view of the Cε3 BC loop. (C) The Lys367 side chain adopts different positions. In the aεFab/IgE-Fc and 8D6 Fab/IgE-Fc complexes, the Lys367 side chains face in opposite directions. Colour code for Figs. S4A, B and C is as follows:1F6A [5] chain B, cyan; 1O0V [6] chain A, salmon; 1O0V chain B, pale green; 2Y7Q [4] chain B, pale orange; 3HA0 [7] chain A, pale blue; 3HA0 chain F, brown; 3H9Y [7]

chain A, gray; 3H9Z [7] chain B, orange; 4EZM [3] chain D, wheat; 4KI1 [8] chain B, pale pink; 4GRG [9] chain C, dark green; 4J4P [2] chain A, blue; 5ANM [10] chain E, magenta; 5ANM chain G, yellow; 5HYS [11] chain G, olive; 5HYS chain K, white; IgE-Fc chain B from the 8D6 Fab/IgE-Fc complex, dark pink. (D) In the 8D6 Fab/IgE-Fc complex, Lys367 is wedged between the two sheets of the Cε3 β-sandwich, partially enclosed in a pocket created by Leu363, Ala364, Val370, His422, His424 and Leu425. Chain B of the 8D6 Fab/IgE-Fc complex is shown. (E) The Cε3 domain C'E and FG loops are positioned further from one another in the 8D6 Fab/IgE-Fc complex (blue) compared with the aεFab/IgE-Fc complex (yellow) [2]. In the 8D6 Fab/IgE-Fc complex, the Lys367 side chain points towards the interior of the Cε3 domain, packing against the BC and FG loops. (F) Lys367 in chain B of the 8D6 Fab/IgE-Fc complex (pink) contacts Asn332 in the chain A Cε2-Cε3 domain linker (yellow).

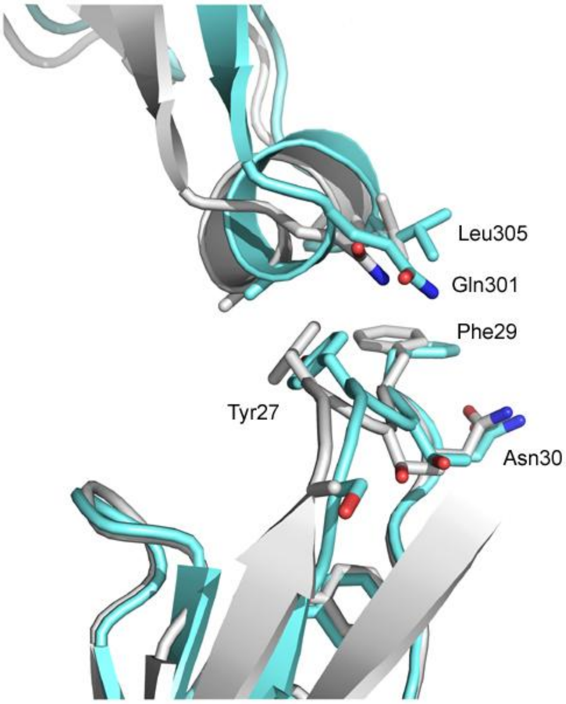

Supplementary Figure S5. Interface between CDRH1 (8D6 Fab heavy chain) and the Cε2 domain. The two IgE-Fc chains in the 8D6 Fab/IgE-Fc complex are not identical. However, at each Fab/IgE-Fc interface, similar contacts are maintained with Gln301, Lys302 and Leu305 (Cε2 domain) as the positions of Gly26-Phe29 (CDRH1) shift. IgE-Fc chain A, and the Fab heavy chain with which it interacts, is coloured cyan, while chain B, and its interacting Fab, is coloured white. Structures were superposed on V_H domain Cα atoms.

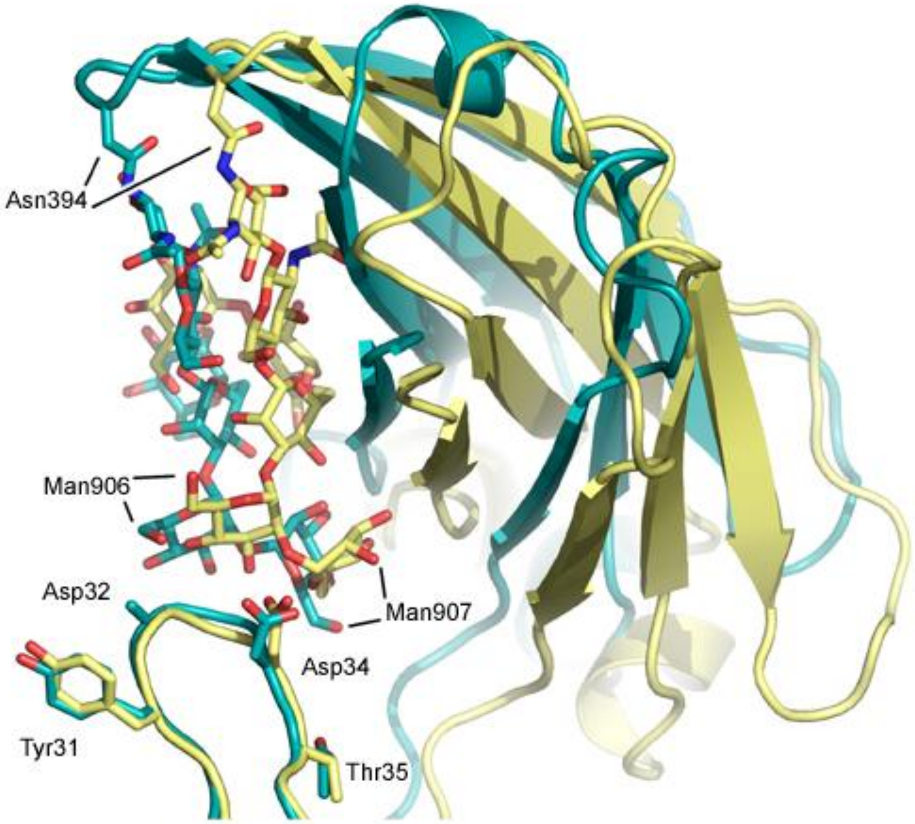

Supplementary Figure S6. The interface between the 8D6 light chain and the oligosaccharide moiety differs for each Fab. For one Fab/oligosaccharide interface (blue), Asp32 and Gly33 (CDRL1) contact Man906 and 907 from the α(1-3) branch, and a hydrogen bond forms between the Asp32 main chain and Man906. For the other Fab/oligosaccharide interface (yellow), Asp32, Gly33 and Asp34 contact equivalent mannose residues, but a hydrogen bond forms between the Asp34 side chain and the α(1-2) glycosidic bond between Man906 and Man907. The Fabs were superposed on V_L domain Cα atoms.

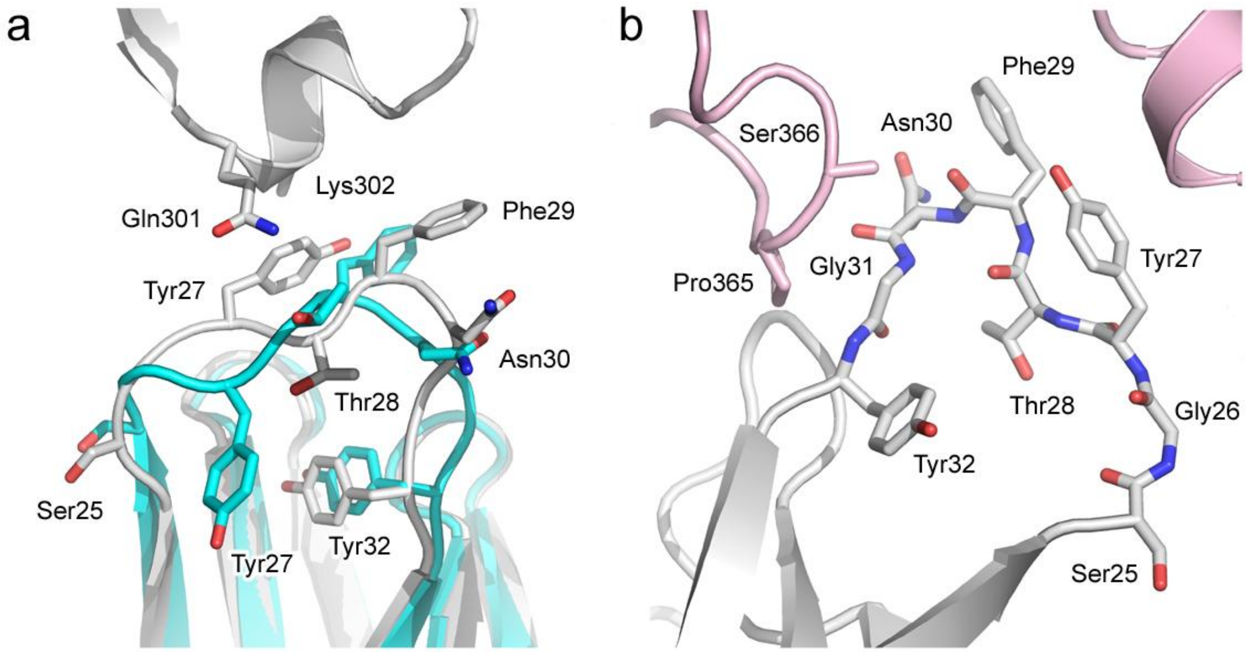

Supplementary Figure S7. Conformational change in the 8D6 Fab. (A) CDRH1 undergoes a conformational change, in which the Tyr27 side chain flips from one face of the main chain in the unbound Fab (blue) to the other (gray) in the 8D6 Fab/IgE-Fc complex, forming an interface with Gln301 and Lys302 (Cε2 domain). Structures were superposed on V_H domain Cα atoms. (B) In the 8D6 Fab/IgE-Fc complex, Pro365 and Ser366 from the Cε3 domain (light pink) contact CDRH1. The conformation adopted by CDRH1 in the unbound Fab structure [(A), blue] is precluded due to steric clashes between Pro365-Ser366, and Asn30-Tyr32.

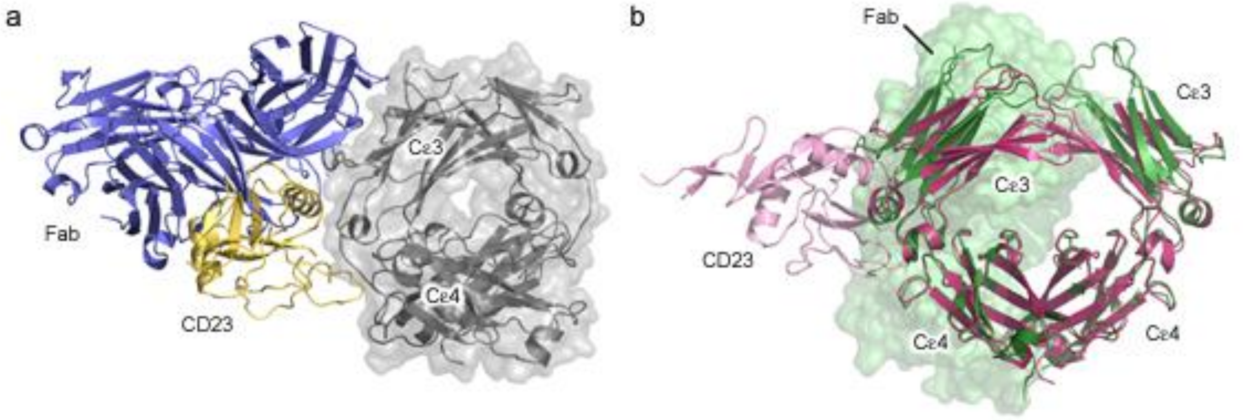

Supplementary Figure S8. Inhibition of CD23 binding to IgE by anti-IgE antibodies. (A) The omalizumab Fab [11,12] (blue) and CD23 [3] (yellow) binding sites on the Cε3 domain overlap. For clarity the Fcε3-4 molecule from the omalizumab Fab/Fcε3-4 complex is not shown. (B) MEDI4212 (pale green) [10] locks the Cε3 domains in an open conformation (dark green), in contrast to the closed conformation (dark pink) found in the CD23/Fcε3-4 complex [3].

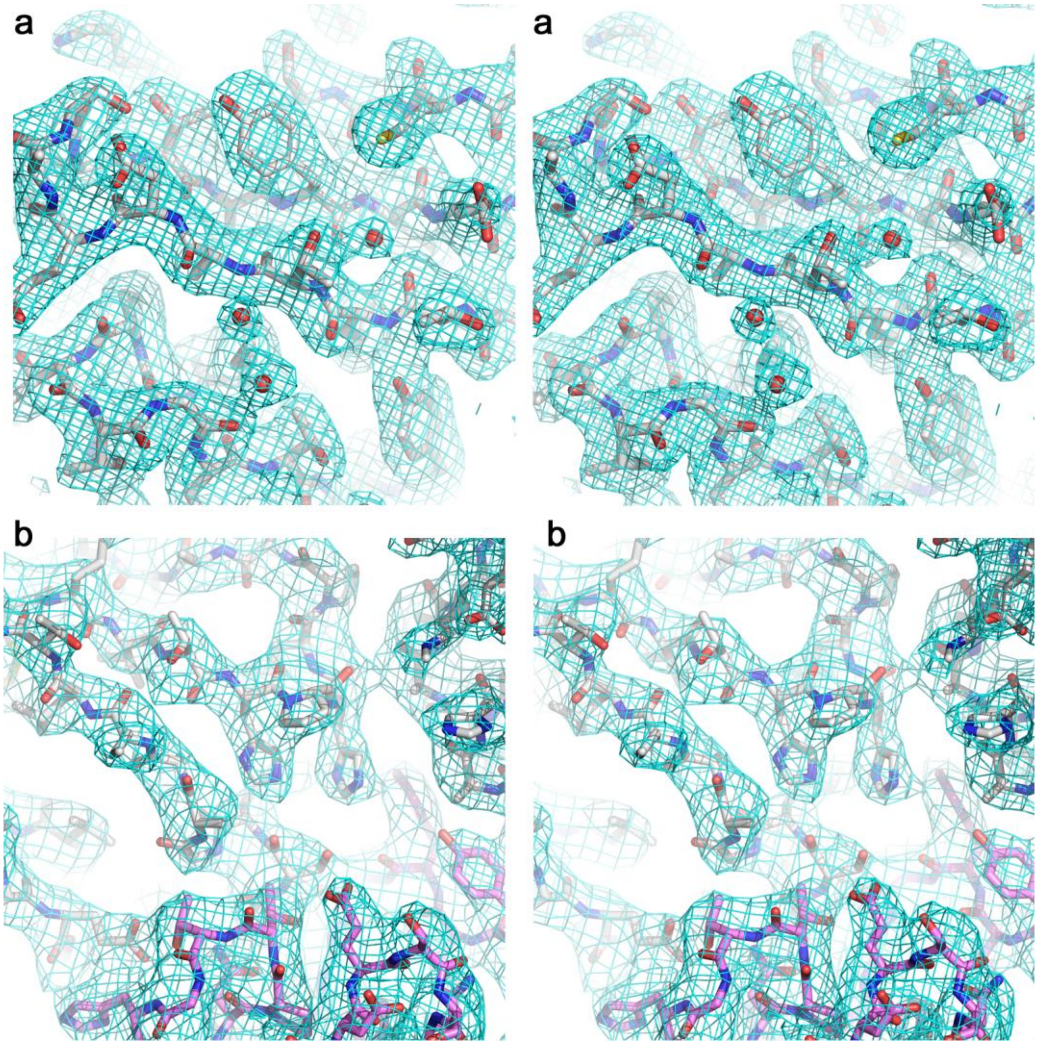

Supplementary Figure S9. Electron density maps. (A) Stereoview of a portion of the 2Fo-Fc electron density map, contoured at 1.0σ, for the 8D6 Fab crystal structure. (B) Stereoview of a portion of the 2Fo-Fc electron density map, contoured at 1.0σ, for the 8D6 Fab/IgE-Fc complex. Carbon atoms for IgE-Fc and the 8D6 Fab are coloured grey and pink, respectively.

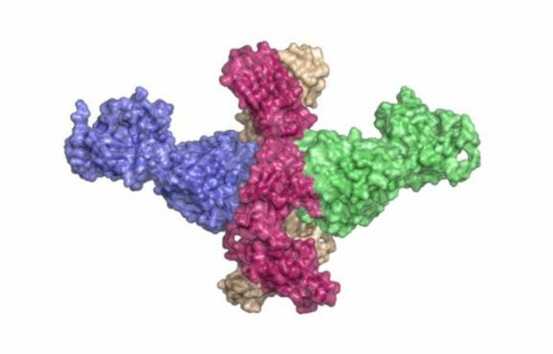

Supplementary Movie S1. Overall structure of 8D6 Fab/IgE-Fc complex. The structure is first rotated 360° about an axis parallel to the pseudo two-fold axis of the Cε2, Cε3 and Cε4 domains, and then rotated 90° towards the viewer. The two IgE-Fc chains are coloured pink and wheat, while the Fabs are coloured green and blue.

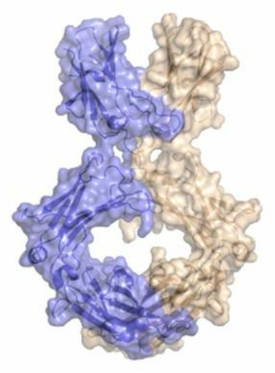

Supplementary Movie S2. Conformational changes in IgE-Fc. The movie shows a morph from the compact, extended IgE-Fc conformation in the 8D6 Fab/IgE-Fc complex, to the extended IgE-Fc conformation captured by aεFab [2], and back to the more compact structure. The morph repeats two times, and depicts the corkscrew motion required to transition between the two structures. The two IgE-Fc chains are coloured blue and wheat.

Supplementary References

1. Myszyka, D.G., Improving biosensor analysis. *J. Mol. Recognit*. **12,** 279-284 (1999).
2. Drinkwater, N., et al., Human immunoglobulin E flexes between acutely bent and extended conformations. *Nat. Struct. Mol. Biol.* **21,** 397-404 (2014).
3. Dhaliwal, B. et al., Crystal structure of IgE bound to its B-cell receptor CD23 reveals a mechanism of reciprocal allosteric inhibition with high affinity receptor FcεRI. *Proc. Natl. Acad. Sci* USA **109**, 12686-12691 (2012).
4. Holdom, M.D. et al., Conformational changes in IgE contribute to its uniquely slow dissociation rate from receptor FcεRI. *Nat. Struct. Mol. Biol*. **18**, 571-576 (2011).
5. Garman, S.C., Wurzburg, B.A., Tarchevskaya, S.S., Kinet, J-P. & Jardetzky, T.S., Structure of the Fc fragment of human IgE bound to its high-affinity receptor FcεRIα. *Nature* **406,** 259-266 (2000).
6. Wan, T., et al., The crystal structure of IgE Fc reveals an asymmetrically bent conformation. *Nat. Immunol*. **3,** 681-686 (2002).
7. Wurzburg, B.A., Jardetzky, T.S., Conformational Flexibility in the IgE-Fc_{3-4} Revealed in Multiple Crystal Forms. *J. Mol. Biol*. **393,** 176-190 (2009).
8. Dhaliwal, B., Pang, M.O.Y., Yuan, D., Beavil, A.J. & Sutton, B.J., A range of Cε3-Cε4 interdomain angles in IgE Fc accommodate binding to its receptor CD23. *Acta Crystallogr. F Struct. Biol. Commun.* **70**, 305-309 (2014).
9. Kim, B., et al., Accelerated disassembly of IgE-receptor complexes by a disruptive macromolecular inhibitor. *Nature* **491,** 613-617 (2012).
10. Cohen, E.S., et al., A novel IgE-neutralizing antibody for the treatment of severe uncontrolled asthma. *mAbs* **6,** 755-763 (2014).
11. Pennington, L.F., et al., Structural basis of omalizumab therapy and omalizumab-mediated IgE exchange. *Nat. Commun*. **7,** 11610 (2016).
12. Davies, A.M., et al., Allosteric mechanism of action of the therapeutic anti-IgE antibody omalizumab. *J. Biol. Chem*. **292,** 9975-9987 (2017).
